# A systematic review of menstrual hygiene management (MHM) during humanitarian crises and/or emergencies in low- and middle-income countries

**DOI:** 10.3389/fpubh.2022.1018092

**Published:** 2022-09-28

**Authors:** Kripalini Patel, Nishisipa Panda, Krushna Chandra Sahoo, Shipra Saxena, Narendra Singh Chouhan, Pratibha Singh, Upasona Ghosh, Bhuputra Panda

**Affiliations:** ^1^Indian Institute of Public Health (IIPH), Public Health Foundation of India (PHFI), Bhubaneswar, Odisha, India; ^2^Health Technology Assessment in India, ICMR-Regional Medical Research Centre, Bhubaneswar, Odisha, India; ^3^United Nations Children's Fund (UNICEF), Bhubaneswar, Odisha, India; ^4^United Nations Children's Fund (UNICEF), New Delhi, India

**Keywords:** menstrual hygiene management, humanitarian crisis, conflict, disaster, pandemic, women health

## Abstract

**Background:**

Poor menstrual hygiene management (MHM) is linked to adverse health, and quality of life, particularly during emergencies. Although in recent times increased emphasis is being laid upon MHM during humanitarian crises—pandemics, disasters and conflicts, the essential components of complete MHM during an emergency are not clearly spelt out. We conducted a systematic review to examine, analyse and describe the existing evidence related to the challenges experienced by women and girls in practicing MHM during humanitarian crises and / or public health emergencies.

**Methods:**

We followed the Preferred Reporting Items for Systematic reviews and Meta-Analyses 2020 guidelines and registered in PROSPERO (CRD42022328636). We searched online repositories: PubMed, Embase, and PsycINFO for articles published between January 2000 and April 2022. For presenting key findings, we used the descriptive statistics and thematic analysis approach.

**Results:**

We identified a total of 1,078 published articles, out of which 78 were selected for a full-text review, and finally 21 articles were included. The pooled prevalence of lack of access to sanitary pads during humanitarian crises was 34 percent (95 percent CI 0.24–0.45). The prevalence of safe and proper sanitary pad disposal practices ranged from 11 to 85 per cent, with a pooled prevalence of 54 per cent (95 per cent CI 0.21–86). Qualitative analyses projected three themes that emerged on MHM during humanitarian crises (1) Availability and affordability of menstrual products, and accessibility to water, sanitation and health (WASH) services, (2) Availability of support system and coping with “period poverty,” and (3) Gender dimensions of menstrual hygiene management. Most studies reported non-availability of MHM products and WASH services during emergencies. Existence of barriers at systemic and personal level posed challenges in practicing menstrual hygiene. Privacy was identified as a common barrier, as emergency shelters were reportedly not women-friendly.

**Conclusion:**

Availability of limited evidence on the subject is suggestive of the need to invest resources for strengthening primary research in low- and middle-income countries and more specifically during emergencies. Context-specific state level policies on MHM during emergencies would help to guide district and sub-district managers in strengthening systems and address barriers for the provision of MHM services during emergencies.

**Systematic review registration:**

https://www.crd.york.ac.uk/prospero/display_record.php?ID=CRD42022328636, identifier CRD42022328636.

## Introduction

Menstrual hygiene management (MHM) has emerged as a major public health concern around the world (1). Poor MHM is not only related to adverse health and psychosocial outcomes but also has social and cultural implications that impair the quality of life amongst women of reproductive age group (2, 3). According to the WHO/UNICEF Joint Monitoring Programme for Water Supply, Sanitation, and Hygiene, women and adolescent girls should adopt a hygienic menstrual management product that can be changed privately. They ought to have access to soap and water for bathing and provisions for disposing of used/ soiled menstrual management products (4). At individual level, there is a need for awareness and integrity—understanding the basic facts about menstrual cycle and how to manage it with dignity, without agony or fear (5). In 2021, Rossouw and Ross coined the phrase “period-poverty” which that connotes lack of access to much-needed hygiene services during periods and adequate facilities to use them, including basic sanitation and menstrual information (6).

Period poverty has been an essential yet underappreciated issue from the viewpoint of reproductive health and rights among women of reproductive age (7). Over 500 million reproductive-age women worldwide lack proper sanitation facilities to manage their menstruation (8). Poor access to menstrual products, non-availability of adequate infrastructure, lack of privacy and disposal facilities, pose critical challenges to MHM (9). Affordability of menstrual products has been a critical issue in high-income countries (10). For example, to combat 'period poverty' Australia abolished the tampon tax in 2019 (10). Furthermore, studies have shown that in low- and middle-income countries (LMICs), such as Kenya, seven percent of women still rely on old clothes and blankets, chicken feathers, mud, and newspaper for MHM. Young females reported having sex with older men to access money for purchasing sanitary pads (11). Similarly, in India, women face significant barriers for menstrual products due to a lack of resources and proper information (12). Beside affordability, menstruation also causes school absenteeism among schoolgirls due to lack of proper facilities at school, as has been the case with Bangladesh (13).

Conflict or war, natural disasters, famine, and disease outbreaks or pandemics can all lead to humanitarian crises. Notwithstanding the ever-increasing emphasis on the MHM needs during public health emergencies and humanitarian crises (14, 15), limited access to safe and private MHM related water, sanitation and hygiene (WASH) facilities during catastrophes continue to pose major challenges for public health experts to overcome (16). However, there is paucity of systematic evidence on MHM challenges during a particular type of emergency, such as, a disaster, a pandemic, and a conflict, or during all types of emergencies. At family level, the health priorities of other family members and children often supersede the menstrual demands of young girls and women. Further, the plight of displaced women and girls in terms of their inability to have sufficient resources (clothes, pads, and underwear) to deal with menstrual is also well-documented. They usually reside in crowded hubs and informal settings with limited toilet facilities and/or private space to change menstrual materials during an emergency (17). Inappropriate disposal of used menstrual materials and improper washing and drying of reusable materials are other significant challenges. These circumstances raise the possibility of compromised health and hygiene, sexual abuse and exploitation in humanitarian contexts (18).

According to a 2012 global review, despite increased attention being paid to MHM in emergencies, there is still lack of clarity on the fundamental components of “full MHM” in an emergency (18). An earlier study cited lack of consistent and adequate preparedness for adopting MHM policies and procedures during humanitarian crises. The COVID-19 pandemic impacted women's health and well-being, particularly MHM (19, 20). The pandemic further exacerbated gender-based violence and led to poor MHM (21–25). There is, however, paucity of systematic evidence on MHM during emergencies. Therefore, we conducted a systematic review to identify and characterize the evidence concerning the difficulties girls and women encounter in maintaining their menstrual hygiene during humanitarian or public health crisis.

## Methods

We followed PRISMA 2020 guidelines for reporting this systematic review (25). We registered this review on PROSPERO (Registration No: CRD42022328636).

### Search strategy, selection criteria, and quality assessment

We adopted a comprehensive search approach relevant to “menstrual hygiene management,” “pandemics,” and “disasters” (Supplementary material). Two authors (KP and NP) independently searched three online repositories: PubMed, Embase, and PsycINFO, to find the studies published between January 2000 and April 2022. First, we performed the primary screening and retrieved articles by screening title and abstract. All the retrieved articles were independently reviewed by other co-authors (BP, UG and KCS). Following the title and the abstract screening, all potentially relevant articles were extracted and evaluated for eligibility through full-text screening. We used the World Bank enlisted countries representing the LMICs; in the full-text review the studies that were not relevant for LMICs were excluded. We also performed a free hand search using google scholar, the reference list of chosen articles and retrieved relevant articles. Any disagreements between authors was settled through mutual discussion.

We included studies focusing on the challenges of maintaining menstrual hygiene among women of the reproductive age during any public health emergency. We set our inclusion criteria as follows: studies published in English language and focused solely on any emergencies such as pandemics, refugee camps, and disasters. Articles on reproductive and sexual health, editorials, commentary, personal views, and review articles were excluded.

We evaluated the study quality using the Mixed Methods Appraisal Tool (MMAT) (26). The quality assessment of chosen studies is described in detail in Supplementary Table 1. All selected studies had specific research question(s) and well-defined data collection techniques. Almost all articles provided thorough findings and maintained coherence between data collection, analysis, and interpretation.

### Data extraction, synthesis, and analysis

Two authors (KP and NP) separately extracted the information using a pre-formed data extraction sheet, and the third author (KCS) cross-checked. The data extraction sheet had the following parameters: author and year, study setting, type of study, type of emergency, study participants, data collection method, data analysis, key results, and recommendations. We used descriptive statistics to present quantitative data based on their characteristics. We estimated the pooled prevalence (meta-analysis) from the available data using MetaXL software Version 5.3. For qualitative data analysis, we used thematic framework analysis (27). We performed the data coding of qualitative information using the MAXQDA software (VERBI Software, Berlin, Germany).

## Results

### Study characteristics

**Figure 1:**
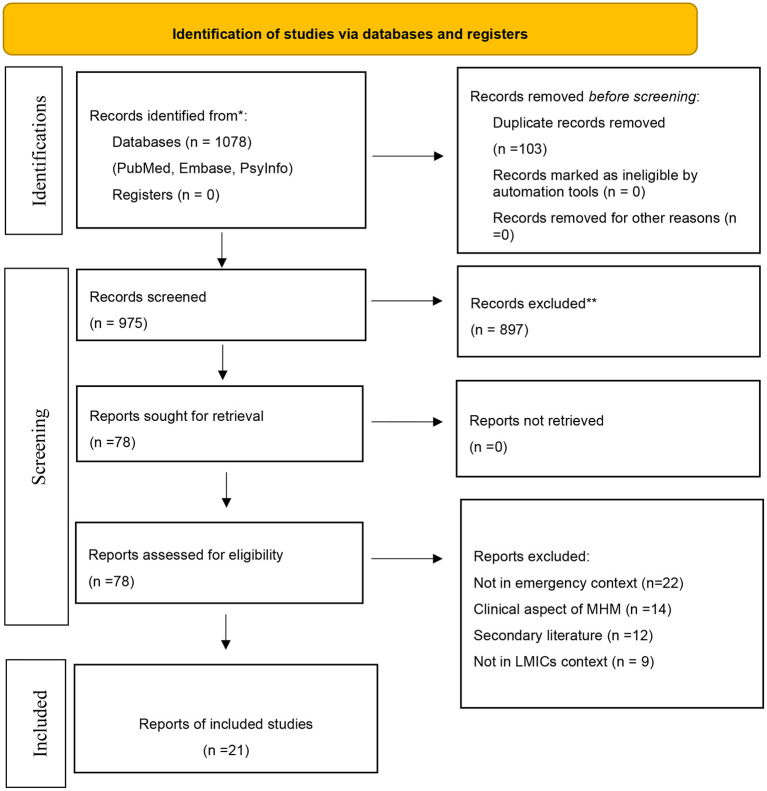
PRISMA flow diagram.

We identified a total of 1,078 articles. Following the title and abstract screening, we selected 78 relevant articles for a full-text review. Following the full-text review, 21 articles were found to be eligible for final inclusion (Figure 1).

In terms of the study design, we observed that out of 21 studies, eight studies adopted a qualitative design, six used a quantitative design, and seven used a mixed-method design. Further, four reported MHM problems associated with pandemics, specifically COVID-19, five were conducted in the context of natural disasters such as floods, cyclones, and earthquakes, and 12 dealt with conflict scenarios among displaced populations in different countries. With regard to the geographies that the studies represented, 11 are found to be from the Indian subcontinent, five are from the Mediterranean and eight are from the African subcontinent. Detailed description of the selected studies is given in Table 1.

**Table 1:** Characteristics of the included studies.

**Author**	**Setting**	**Humanitarian crises**	**Study design**	**Participants**	**Data collection method**	**Analysis method**	**Major topic discussed**
Garg et al. (22)	India	Pandemic	Quantitative	Students	Survey (*n* = 1,371)	Descriptive	Access and affordability
Garg et al. (23)	India	Pandemic	Quantitative	Students	Survey (*n* = 1,371)	Descriptive	Access to sanitary pad
Ciardi Sassone et al. (28)	India	Pandemic	Quantitative	Adolescent girls	Survey (*n* = 50)	Descriptive	Menstrual product
Hensen et al. (29)	Zambia	Pandemic	Mixed method	Adolescent girls and women	FGD (*n* = 2), Survey (*n* = 6,374)	Thematic analysis	Access to menstrual product
Maknun et al. (30)	Bangladesh	Disaster (flood)	Mixed method	Adolescent girls, women, local volunteer, and medical staff	Survey (*n* = 100), FGDs (*n* = 3), KIIs (*n* = 5)	Not Mentioned	Knowledge, practice of MHM, and WASH facility
Bhattacharjee (31)	India	Disaster (flood)	Qualitative	Women and adolescent girl	IDIs, FGDs (*n* = 84)	Not Mentioned	Access to sanitary pad and infrastructure
Krishnan and Twigg (32)	India	Disaster (flood and cyclone)	Qualitative	Women and adolescent girls, NGO staff and experts, and government officials	FGDs (*n* = 14), IDIs (*n* =1 6)	Thematic analysis	Overall MHM
Downing et al. (33)	Vanuatu	Disaster (cyclone)	Mixed method	Women and girls	FGDs (*n* = 12), KIIs (*n* = 2), Survey (*n* = 136)	Framework analysis	Access to sanitary products, water supply and privacy
Budhathoki et al. (34)	Nepal	Disaster (earthquake)	Mixed method	Women and girls	IDIs (*n* =1 17)	Thematic analysis	Menstrual absorbent
Rakhshanda et al. (35)	Bangladesh	Conflict	Mixed method	Adolescent girls and their mothers	Survey (*n* = 340), IDI (*n* = 7), FGD (*n* = 2)	Descriptive and thematic	Knowledge, practice, and associated factor for MHM
Kemigisha et al. (36)	Uganda	Conflict	Mixed method	Adolescent girl	IDIs, Survey (*n* = 260)	Descriptive and thematic	Menstrual absorbent
Soeiro et al. (37)	Brazil	Conflict	Quantitative	Adolescents and young women	Survey (*n* = 167)	Descriptive	Not received hygiene kit
Schmitt et al. (18)	Myanmar, Lebanon	Conflict	Qualitative	Humanitarian staff, adolescent girls and women	KII (*n* = 17), FGD (*n* = 2)	Thematic analysis	Lack of access to privacy
Schmitt et al. (38)	Bangladesh	Conflict	Qualitative	Humanitarian response staff, adolescent girls and women	KII (*n* = 19), FGD (*n* = 2), observation of facilities (*n* = 8)	Thematic analysis	Menstrual disposal
Krishnan and Twigg (39)	India	Conflict	Mixed method	Women and stakeholders	Survey (*n* = 374), KIIs (*n* = 40)	Descriptive analysis	WASH
Ivanova et al. (40)	Uganda	Conflict	Qualitative	Adolescent girls	IDI (*n* =2 8), FGD (*n* = 2)	Thematic analysis	Menstrual management
Metusela et al. (41)	Multiple countries	Conflict	Qualitative	Women	IDI (*n* = 84), FGD (*n* =1 6)	Thematic analysis	Knowledge on menstruation
Calderón-Villarreal et al. (42)	Bangladesh, Kenya, Uganda, Sudan, Zimbabwe	Conflict	Quantitative	Refugee households	Survey (*n* = 5,632)	Descriptive	WASH
Rocha et al. (43)	Brazil	Conflict	Quantitative	Women aged 18–49 years	Survey (*n* = 177)	Descriptive	Menstrual product
Korri et al. (44)	Lebanon	Conflict	Qualitative	Adolescent girls	FGDs (*n* = 8)	Thematic analysis	MHM experience and
Majed and Touma (45)	Lebanon	Conflict	Qualitative	Refugee women and girls	FGDs (*n* = 10), IDIs (*n* = 38)	Not Mentioned	Menstrual practices

**Figure 2:**
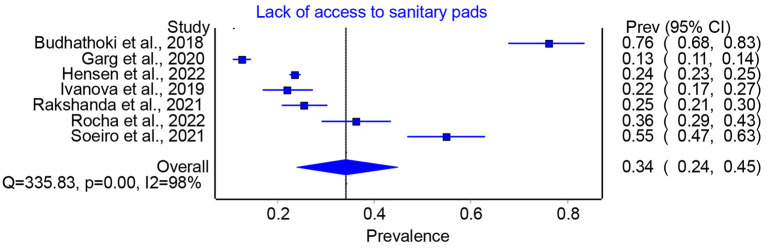
Prevalence of lack of access to sanitary pads in humanitarian crises.

**Figure 3:**
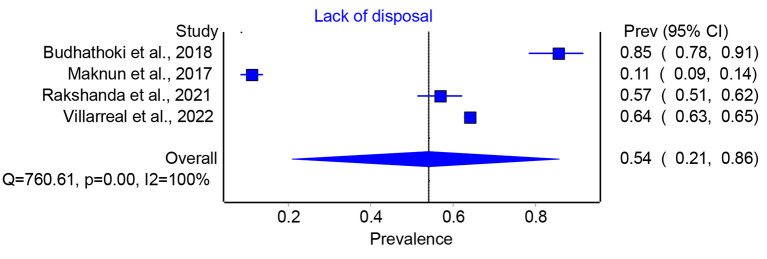
Prevalence of lack of disposal facilities in humanitarian crises.

According to the data, the total prevalence of lack of access to sanitary pads in humanitarian crises ranged from 13 to 76 per cent, with the pooled prevalence of 34 per cent (95 per cent CI 0.24–0.45) (Figure 2). Similarly, the prevalence of appropriate disposal of sanitary pad ranged from 11 to 85 per cent, with a pooled prevalence of 54 per cent (95 per cent CI 0.21–86) (Figure 3). Thus, the findings showed a significant lack of access to sanitary pads and their disposal, making MHM difficult during crises.

Three themes emerged from the analysis of qualitative data: (1) Availability and affordability of menstrual products, and accessibility to WASH services, (2) Availability of support system and coping with “period poverty” during humanitarian crisis, and (3) Gender dimensions of menstrual hygiene management during humanitarian crises. The qualitative data on MHM focused on challenges on availability, accessibility, and affordability during various humanitarian crises such as pandemics, disasters and conflicts. Table 2 summarizes major barriers to MHM during humanitarian crises / emergencies.

**Table 2:** Major challenges in managing menstrual hygiene during humanitarian crises.

**Challenges**	**Pandemic**	**Disasters**	**Conflicts**
Period products	Lack access to basic materials, such as sanitary pads, cloths, and underwear Used rags, piece of saree, old clothes, piece of cotton	Lack of availability of menstrual products Difficulties to sudden shift to pads Lack of sufficient distribution of period products Lack of quality in period products Not received any hygiene kit Lack of access to clean underwear Lack of affordability because of meager household income Uses old cloths/rags because of lack of affordability	Lack of availability of quality sanitary products Lack of affordable disposable sanitary products and soaps The emergency kits provided lacked sufficient sanitary pads Irregular supply of sanitary kits
WASH	Not reported	Used flood water for cleanliness Lack of access to laundry facilities Lack of water source inside the latrines Lack of gender segregated washrooms Threw the used menstrual/blood-stained cloth without washing New shelters provide few latrines which leads to long lines and discomfort Scarcity of water in displacement settings Ponds become inaccessible during floods Difficulties in access to a toilet for changing pads Shelter in community centers—toilets are not always accessible	Lack of water supply in general and also near latrines Lack of gender segregated washrooms and shared bathrooms were crowded, filthy and lacked a separate dumping area Lack of washroom/bathroom Access to latrines in night was difficult due to poor lighting and pathways
Privacy	Not reported	Absence of a private and safe place Visible wide breaches in the bamboo walls Lack of locks on the doors Change their period products outdoors Lack of private places to dry clothes Women had to travel long distances before sunrise or late in the night Lack of privacy for personal hygiene, while washing or disposing of pads Lack of access to private locations to change menstrual products, hence carrying pad and walking far from the location to change pad Lack of private locations within their shelters	Lack of private locations for women to wash, dry and change their menstrual products Lack of locks on the doors of washrooms
Disposal	Not reported	Lack of availability of trash cans within the restrooms Worried about gender-sensitive washrooms—dispose of used pads difficult	Lack of availability of dustbins for disposal of used sanitary products
Information	Lack of knowledge on menstruation before menarche	Lack of access to adequate information and knowledge Lack of knowledge of menstruation or frequency of changing pads Lack of information about how they would dispose of used pads	Lack of access to adequate information and knowledge

### Theme 1. Availability and affordability of menstrual products, and accessibility to WASH services

#### During pandemic

According to studies, the availability of and accessibility to adequate menstrual hygiene products was a substantial concern during the pandemic (22, 23, 28, 29). As per a study in Zambia, during the COVID-19 pandemic, adolescent girls and young women were compelled to wear use clothes as pads because they could not access sanitary pads (29). This study also showed that the availability and use of menstrual products were reduced in the post-pandemic period compared to the pre-pandemic period (29).

“*We [adolescent girls] suffered because the hubs were closed, and there were no sanitary pads available. We were forced to wear old clothes”* (29).

During the COVID-19 pandemic, financial constraints hampered access to menstrual products among women and girls. According to some studies in India, purchasing sanitary pads during a pandemic was influenced by factors such as the job loss or inadequate income (22, 23, 28). For example, those have with a monthly family income of INR 25,000 or less and those with a monthly family income of INR 25,000–50,000 (AOR 0.560, 95 per cent CI: 0.334–0.937) have had difficulties in purchasing sanitary pads during pandemic (22).

#### During natural disasters

During a natural disaster, menstrual hygiene products, WASH services, and privacy were compromised. According to a survey conducted in Bangladesh, 56 percent of women from low socioeconomic strata utilized old clothes as absorbent during menstruation during a disaster. At the same time, 15 per cent did not use any product due to scanty blood flow (30). In Nepal, girls and women faced difficulties to access menstrual hygiene supplies during the devastating earthquake in 2015 (35). Closure of roads and stores in the aftermath of a disaster impacts the availability of menstruation supplies (33). A study in India found that women experienced double burden of poor access to sanitary pads and inadequate availability of water to wash used clothes during emergencies; consequently, they discarded the used clothes instead of reusing it (31). It was also observed that in post-flood situation, prices of sanitary pads rise as supply falls (30); in such scenario, women who use sanitary pads opt for the lowest-priced product available, compromising with the quality.

With regard to waste disposal, several studies reported difficulties in disposal of used menstrual products (30, 33). A study from Bangladesh explained that 23 percent of the women had difficulties in cleaning their used menstrual clothes during the flood time; as a result, the disposal rates increased during a disaster (30). Another study from the Republic of Vanuatu stated that women were worried about the disposal of used sanitary pads, especially if they had to share toilets with men (33).

#### During conflict situations

Many studies reveal the lack of WASH services and menstrual products was a significant problem during conflicts for out-migrants and displaced population living in camps at unfamiliar location (32, 36, 40, 43). To manage their menstruation, women used reusable cloth due to unavailability and affordability of sanitary pads (15). Water availability for drinking, bathing, and washing was another barrier due to lack of availability of water inside toilets. Access to toilets at night was also an issue in the camps due to low illumination on routes and in bathrooms and worries of sexual violence and harassment (18, 38, 42).

In conflict contexts, lack of affordable menstrual products adversely affects girls' and women's menstrual hygiene (18, 35–44). Many Syrian refugees in Lebanon said they preferred disposable pads but those were expensive (18). In refugee settings, women couldn't afford soap, so they washed their hands with water or ashes and reused absorbents with water (35). In many displaced situations, women received menstrual hygiene kits but faced difficulties in using them, as kits were typically distributed without instructions on how to use them. Insufficient distribution of sanitary pads (35), MHM kits, and irregular supply of relief kits among displaced girls and women (18) were critical challenges in conflict situations.

Eight out of 13 studies further explained women's lack of access to adequate information on MHM during conflicts. According to a study conducted in a refuge setting of Bangladesh, women did not change their clothes, rags, or pads every 4 h since they were unaware of the need (36). Similarly, studies found that girls lacked adequate knowledge on the frequency of changing their pads and its disposal method (35, 36, 39, 40). Participants also noted the problems they had while attempting to use disposable pads efficiently for the first time because of insufficient guidance (44). Due to lack of space and bin for disposal of menstrual products, some bury or flush their used pads (35, 45).

“*During the night, I flush the pads in the public toilet. It chocks when everyone flushes*.”

### Theme 2. Availability of support system and coping with “period poverty” during humanitarian crisis

During humanitarian emergencies, many young women experience “period poverty.” They could not obtain or buy menstrual health items to meet their monthly requirements. Furthermore, lack of privacy compelled them to engage in maladaptive behavior, which harmed their mental health and dignity.

Due to limited income during COVID-19 lockdown, women prioritized other household needs over purchasing sanitary pads (22). Similarly, in Zambia adolescent girls and young women revealed their inability to afford sanitary pads during COVID-19 pandemic, making them manage menstruation with pieces of cloth (29). Participants in flood-prone areas proposed modifying floating toilet facilities which was constructed of banana trees, and utilized for cleanliness (30). Another study in India indicated that as a response to humanitarian circumstances, MHM display kiosks were set up where women were taught the use of MHM products available in the hygiene kit and awareness raised on menstrual hygiene (32). Many studies found that despite the availability of limited number of single rooms in the camps, some families had built small washrooms to access safe sanitation (18, 38). In cases where low purchasing power leads to inadequate stock of menstrual materials, females were asked to use tissue paper as an alternative (45).

In a Bangladeshi refugee camp, laundry facilities had many open washing booths with a shower curtain divider between each stall to allow ladies an added degree of privacy when cleaning compassionate things (38). Similarly, immediately after the floods, humanitarian organizations-built privacy screens made of tarpaulin sheets near the walls of washrooms for displaced residents (39). Furthermore, assisting one another through resource sharing was common among female refugees: they shared physical resources while respecting each other's dignity (15). Studies revealed that they constructed new waste disposal systems in response to crises at hospitals and refugee camps (38). Although women received sanitary pads and hygiene kits from local humanitarian organizations, lack of culturally suitable sanitary products hampered its adoption by the communities; therefore, they continued to use clothes or rags (31). A study conducted in Vanuatu during a storm found that participants expressed discomfort about receiving MHM kits and other non-food goods. In contrast, others described receiving items that were foreign to them and being confused about how to utilize the product (33).

### Theme 3. Gender dimensions of menstrual hygiene management during humanitarian crises

The emergency shelters were not women-friendly in Zambia; thus, women's security, privacy, and health requirements were mainly overlooked (30). During displacement, girls did not use washroom facilities due to presence of boys and men in and around the camp. According to a study of Syrian girls and women, shared bathrooms in displacement camps were crowded, unclean, and lacked a waste disposal space. They also cited lack of gender segregated toilets, significant space between the bamboo walls permitting vision, and lack of door latch compromising security (18). Similarly, the absence of safe and private areas to change menstrual products was a significant problem, as community toilets were typically outside tents, forcing menstruating women to carry menstrual products across the camp (45). Moreover, many toilets lacked a latch because of which women felt insecure during changing menstrual product.

“*You don't feel like you are in a bathroom, when you use the latrines. It feels like you are in the open air*” (45).

Women had difficulty locating private spaces within their shelters to change, wash, and dry their reusable clothes (18, 39). As a result, they had to use their menstrual products for long hours comprising hygiene and could change it while going for open defecation or bathing near water bodies or shrubs in the early morning or late at night (39, 44).

“*The tent walls are often made of transparent plastic sheeting. Someone from the outside can see you in there*” (44).

Besides, access to water points was difficult in flood-prone areas, limiting women's and girls' privacy in washing their menstrual linen near water sources (because the blood washed into open drains was visible to others) forcing women to travel long distances in the dark (before sunrise or late at night) to cater to their personal needs (39). Recognizing the need for more water during menstruation and the scarcity of water in disaster scenarios, many women were concerned about receiving a negative response from men if they were spotted using extra water (33).

“*If we wish to wash our blood-stained underwear and clothing, some men may argue that we are wasting water and that it is an emergency that we manage our water resources*” (33).

Another critical problem experienced by refugee women was the inability to purchase menstrual products if sold by male members at the store. Thereby, unaffordability and scarcity are not the only reasons deterring women from purchasing sanitary pads; the gender of the shop owner is also important. Usually, even in an emergency, women are uncomfortable in purchasing or discussing their MHM-related needs with male-sellers of sanitary product and male doctors.

“*We make several trips until there was a woman serving, so we could buy products comfortably*” (30).

The level of education and occupation of fathers in the family are found to influence “affordability” and “accessibility” to sanitary products, especially in LMICs where female unemployment is relatively high. A study conducted in India revealed that students with college-educated fathers were less likely (AOR: 0.559, 95% CI: 0.349–0.897) to experience difficulty in gaining access to sanitary pads than those with high school-educated fathers. On the other hand, students whose fathers were farmers were more likely (AOR: 1.998, 95% CI: 1.013–3.937) to have difficulty in obtaining sanitary napkins than students whose fathers were government officials (23).

## Discussion

The systematic review reveals that humanitarian crises further more exacerbated 'period poverty'. During emergencies, inadequate availability of and poor access to MHM products, WASH services, privacy, and disposal mechanisms have been identified as common impediments. The emergency shelters were not women-friendly, lacked WASH services, and, wherever they existed, were not in useable condition or catered to the MHM needs of women and girls. Value-added interventions were reported to have been implemented in various settings with some success. For instance, education on menstrual hygiene, distribution of emergency hygiene kits, design of a new waste disposal system, and installation of a hub as a private space for MHM practices were all implemented with varying degree of success to ensure menstrual hygiene during an emergency.

It is well-documented that during humanitarian crises, the safe management of WASH services is vital. It is equally true that the immediate aftermaths of emergencies include supply chain break-down, loss of life and property, mental trauma and stress and panic buying, which undermine the continuity and quality of ordinary MHM processes (8, 46). Therefore, it is critical to ensure that MHM supplies and WASH services are put in place during emergencies to alleviate “period poverty” by providing menstrual supplies and basic WASH services at community level, camps and institutions (47). It is advised to timely stock hygiene kits and sanitary items and provide them to girls and women in times of need (48, 49). The United Nations Women, through the Council of Governors, had provided dignity kits to women and girls in COVID-19 quarantine sites in Kenya, targeting the countries afflicted by the pandemic (50). The primary reason women and girls leave their shelters at night was to use the toilet. Interestingly, a study in Uganda found that kerosene lamps made refugee women in camps feel safer (51). Other studies have shown that a successful MHM response in humanitarian circumstances requires appropriate lighting for latrine use at night (52). Therefore, enabling access to safe and gender appropriate WASH services inclusive of the provision of lights and door-closures are essential for privacy and safety of vulnerable women and girls (51, 52).

Despite the progressive sensitization of world community and policy makers about the need for having a robust MHM policy at every state level and disaster-specific MHM plan at district level, the progress made on this front is very limited. Many gaps continue to glare at public health experts and programme managers. First, uniformly decided indicators needs to be developed to monitor and evaluate MHM interventions; second, consensus needs to be built around the basket of services defining essential and comprehensive MHM services and resources in various emergency settings; third, roles and responsibilities of key departments and actors should be delineated and integrated with the existing framework of service delivery; fourth, clear guidelines for assessing the context and the needs of beneficiaries need to be developed and institutionalized (53). Existing systematic reviews on the social impact of MHM reveal the role of educational interventions in enhancing MHM practices (2, 54); therefore, the district administration needs to prioritize capacity building of service providers and beneficiaries on the components of MHM, and strategies to meet MHM needs during emergencies.

It is essential for women and girls to have access to free and fair menstrual products and safe services for use under extreme circumstances, including emergency. Local governments and knowledge partners could play a significant role in mapping out micro-plans for the geographies of their jurisdiction, keeping in mind the local cultural practices, sources of water and sanitation, infrastructural arrangements in various settings, and climate disasters that frequently hit the region. Women and girls must have access to safe and suitable facilities with access to WASH for changing and cleaning their menstrual products and disposing them safely (48). The plan should include availability of feminine hygiene products in its list of health services and essentials (55). Integrating MHM into the overall pandemic response system and the toolbox developed in collaboration with the global humanitarian response community are a few such examples of unique MHM services provided during emergencies. The toolkit includes practical information and tools for MHM programming planning, implementation, and monitoring (56–58). Moreover, during an emergency, availability of a well-formulated MHM strategy and preparedness of the public distribution system in LMICs, inclusive of the Indian subcontinent, the Mediterranean, and the African subcontinent could play a crucial role in mitigating the acute needs of women, especially school going girls and housewives. International organizations—the UNICEF, the WHO, and other multi-lateral agencies—ought to provide hand-holding support for development of an effective MHM policy and plan at national and sub-district levels.

Gender inequality, discriminatory social norms, cultural taboos, poverty, and lack of essential services often result in unmet menstrual health and hygiene demands. Emergencies can exacerbate the “period poverty” by restricting mobility, liberty, and opportunities, compromising education, and causing stress and anxiety. Hence, gender-transformative WASH services is critical in general and during emergencies to fight against “period poverty” in particular (59, 60). This technique empowers vulnerable women and girls to speak up, lead, and develop movements. To confront and transform prevailing social, economic, and political structures that perpetuate gender inequity—men and boys must be partners and champions of change to fight against period poverty (61, 62).

Transformative approaches understand how gender disparities intersect and intensify other injustices for better programming. Women's empowerment is a complicated, multi-layered element of gender transition. Women's absence from WASH policies and programmes must be studied, addressed, and altered. WASH professionals could learn from gender specialists and campaigns that have promoted gender-transformative change by confronting gender norms and stereotypes (63, 64). In many cultures, traditional leaders have a lot of influence over social norms. They are key 'gatekeepers' who spread ideas and information to communities. Working with traditional leaders can help change sticky gender stereotypes and promote gender-transformative WASH interventions for better MHM during emergency situation.

Although, we conducted a systematic review of all research studies relevant to MHM in an emergency environment, we excluded papers dealing with MHM in a non-emergency context. We attempted to create a search strategy using every available phrase. However, the search technique was restricted to documents published in English only due to linguistic constraints. We ignored similar articles that were published in other languages.

## Conclusion

In emergencies, it has been reported that MHM products and WASH facilities are mostly unavailable. Where available, privacy and security has been identified as a common barrier—the emergency shelters were not women-friendly, impeding the fundamental rights of women during emergency. The results reveal insufficient data to guide policymakers and programme managers about context-specific interventions, suggesting that additional primary research and a country-specific emergency MHM preparedness plan are required.

## Data availability statement

The original contributions presented in the study are included in the article/Supplementary material, further inquiries can be directed to the corresponding author/s.

## Author contributions

KP, NP, and KS conducted literature review, extracted and undertook content analysis, and prepared first draft of manuscript. UG provided critical inputs to finalizing search strategy and data collection tools and reviewed the protocol. SS, NC, and PS reviewed and strengthened various sections of the manuscript. BP revised and finalized the manuscript. All authors contributed to the article and approved the submitted version.

## Funding

This study was funded by the United Nations Children's Fund (UNICEF), Bhubaneswar, India PD Ref No: INDO/2021/017. This study is a by-product of the project on Menstrual health and hygiene – situation analysis and strategic roadmap in Odisha.

## Conflict of interest

Author SS, NC, and PS were employed by United Nations Children's Fund (UNICEF). The remaining authors declare that the research was conducted in the absence of any commercial or financial relationships that could be construed as a potential conflict of interest.

## Publisher's note

All claims expressed in this article are solely those of the authors and do not necessarily represent those of their affiliated organizations, or those of the publisher, the editors and the reviewers. Any product that may be evaluated in this article, or claim that may be made by its manufacturer, is not guaranteed or endorsed by the publisher.
