# Supplementary material for: A systematic review of menstrual hygiene management (MHM) during humanitarian crises and/or emergencies in low- and middle-income countries
Source: Front Public Health. 2022 Sep 28;10:1018092. doi: 10.3389/fpubh.2022.1018092 (PMC9555566; doi:10.3389/fpubh.2022.1018092)
Supplement: Supplementary file 1 [file Table_1.docx]

| **Qualitative studies** | | | | | | | |
| --- | --- | --- | --- | --- | --- | --- | --- |
| References | S1 | S2 | 1.1 | 1.2 | 1.3 | 1.4 | 1.5 |
| Bhattacharjee, 2019 [31] | No | Yes | Yes | Yes | Yes | No | Yes |
| Sneha Krishnan (2016) | Yes | Yes | Yes | Yes | Yes | Can't tell | Yes |
| Schmitt et al., 2017 [18] | Yes | Yes | Yes | Yes | Yes | Yes | Yes |
| Schmitt et al., 2021 [38] | Yes | Yes | Yes | Yes | Yes | Yes | Yes |
| Korri et al., 2021 [44] | Yes | Yes | Yes | Yes | Yes | Yes | Yes |
| Majed & Touma, 2020 [45] | Yes | Yes | Yes | Yes | Yes | Yes | Yes |

**Table 1.** Quality Assessment of the studies using the Mixed Methods Appraisal Tool (MMAT)

| **Quantitative studies** | | | | | | | | | | | | | |
| --- | --- | --- | --- | --- | --- | --- | --- | --- | --- | --- | --- | --- | --- |
| References | | S1 | | S2 | | 3.1 | | 3.2 | | 3.3 | | 3.4 | 3.5 |
| Ciardi Sassone et al., 2022 [28] | | Yes | | Can't tell | | No | |  | | Yes | |  |  |
| Garg et al., 2020a [22] | | Yes | | Yes | | Yes | | Yes | | Yes | | Yes | Yes |
| Garg et al., 2020b [23] | | Yes | | Yes | | Yes | | Yes | | Yes | | Yes | Yes |
| Soeiro et al., 2021 [37] | | Yes | | Yes | | Can't tell | | Yes | | Can't tell | | Can't tell | Yes |
| Calderón-Villarreal et al., 2022 [42] | | Yes | | Yes | | Yes | | Yes | | Yes | | Yes | Yes |
| Rocha et al., 2022 [43] | | Yes | | Yes | | Can't tell | | No | | Yes | | Can't tell | Yes |
| **Mixed Method studies** | | | | | | | | | | | | | |
| References | S1 | | S2 | | 5.1 | | 5.2 | | 5.3 | | 5.4 | | 5.5 |
| Rakhshanda et al., 2021 [35] | Yes | | Yes | | No | | Yes | | Yes | | Can’t tell | | Yes |
| Maknun et al., 2017 [30] | Yes | | Yes | | No | | Yes | | Yes | | Can’t tell | | Yes |
| Downing et al., 2021 [33] | Yes | | Yes | | Yes | | Yes | | Yes | | Can’t tell | | Yes |
| Budhathoki et al., 2018 [34] | Yes | | Yes | | Yes | | Yes | | Yes | | Can’t tell | | Yes |
| Ivanova et al., 2019 [40] | Yes | | Yes | | No | | Yes | | Yes | | Can’t tell | | Yes |
| Krishnan & Twigg, 2016 [39] | Yes | | Yes | | Yes | | Yes | | Yes | | Yes | | Yes |
| Kemigisha et al., 2020 [36] | Yes | | Yes | | Yes | | Yes | | Yes | | Can’t tell | | Yes |
| Hensen et al., 2022 [29] | Yes | | Yes | | Yes | | Yes | | Yes | | Yes | | Yes |
